# Poroid Hidradenoma: A Rare Finger Lesion

**DOI:** 10.7759/cureus.68381

**Published:** 2024-09-01

**Authors:** Jana N Satma, Tara Chadab, Marina Landa, Ryan Engdahl

**Affiliations:** 1 Internal Medicine, St. George's University School of Medicine, Saint George, GRD; 2 Department of Surgery, Division of Plastic and Reconstructive Surgery, New York Presbyterian, The University Hospital of Cornell and Columbia, New York, USA; 3 Pathology, Harlem Hospital Center, New York, USA; 4 Surgery, Harlem Hospital Center, New York, USA

**Keywords:** finger, hand, lesion, tumor, dermal, adnexal, eccrine, poroma, poroid hidradenoma

## Abstract

Poroid hidradenomas are uncommon tumors originating from eccrine sweat glands, often presenting as dermal nodules predominantly composed of cuticular and poroid cell types. They belong to the broader category of poroid neoplasms, which include hidroacanthoma simplex, dermal duct tumor, and eccrine poroma. Despite their rarity, poroid hidradenomas require accurate diagnosis and appropriate management due to potential overlaps in their histological features with other adnexal tumors, including digital papillary adenocarcinoma. We report a case of a 56-year-old female presenting with a nodular lesion on the palmar aspect of her right middle finger, which was excised under local anesthesia. Histopathological examination confirmed the diagnosis of poroid hidradenoma, highlighting the importance of maintaining a high clinical suspicion and precise histological evaluation in managing such an uncommon dermatological condition of the finger.

## Introduction

Hidradenomas are tumors originating from sweat glands and are classified into two main types. One type arises from eccrine sweat glands, presenting as dermal nodules disconnected from the overlying epidermis and primarily composed of cuticular and poroid cell types, categorized as "poroid hidradenomas." The second type exhibits apocrine differentiation, composed of mucinous, polygonal, and clear cells [1]. Poroid neoplasms are categorized into four subtypes based on the location of neoplastic cells, such as hidroacanthoma simplex, dermal duct tumor, poroid hidradenoma, and eccrine poroma. The poroid hidradenoma remains the least documented variant as it is extremely rare [2].

Hidradenoma has been described as a benign tumor exhibiting eccrine characteristics. Its typical presentations include a single tender nodule with a diameter ranging from 0.5 to 2 cm [3]. The lesion classically appears skin-colored or slightly reddish, but cystic areas may impart a bluish hue. It consists of both solid and cystic components and remains confined within the dermis. Poroid hidradenoma is commonly treated with resection. However, Mohs surgery has shown efficacy in treating large and recurrent cases [4]. There is only one other report in the literature citing this diagnosis as a finger lesion [5]. This case report aims to provide further insights into the clinical characteristics, diagnostic challenges, and management strategies associated with poroid hidradenoma. It highlights the importance of accurate diagnosis and appropriate treatment for such uncommon dermatological conditions.

## Case presentation

**Figure 1:**
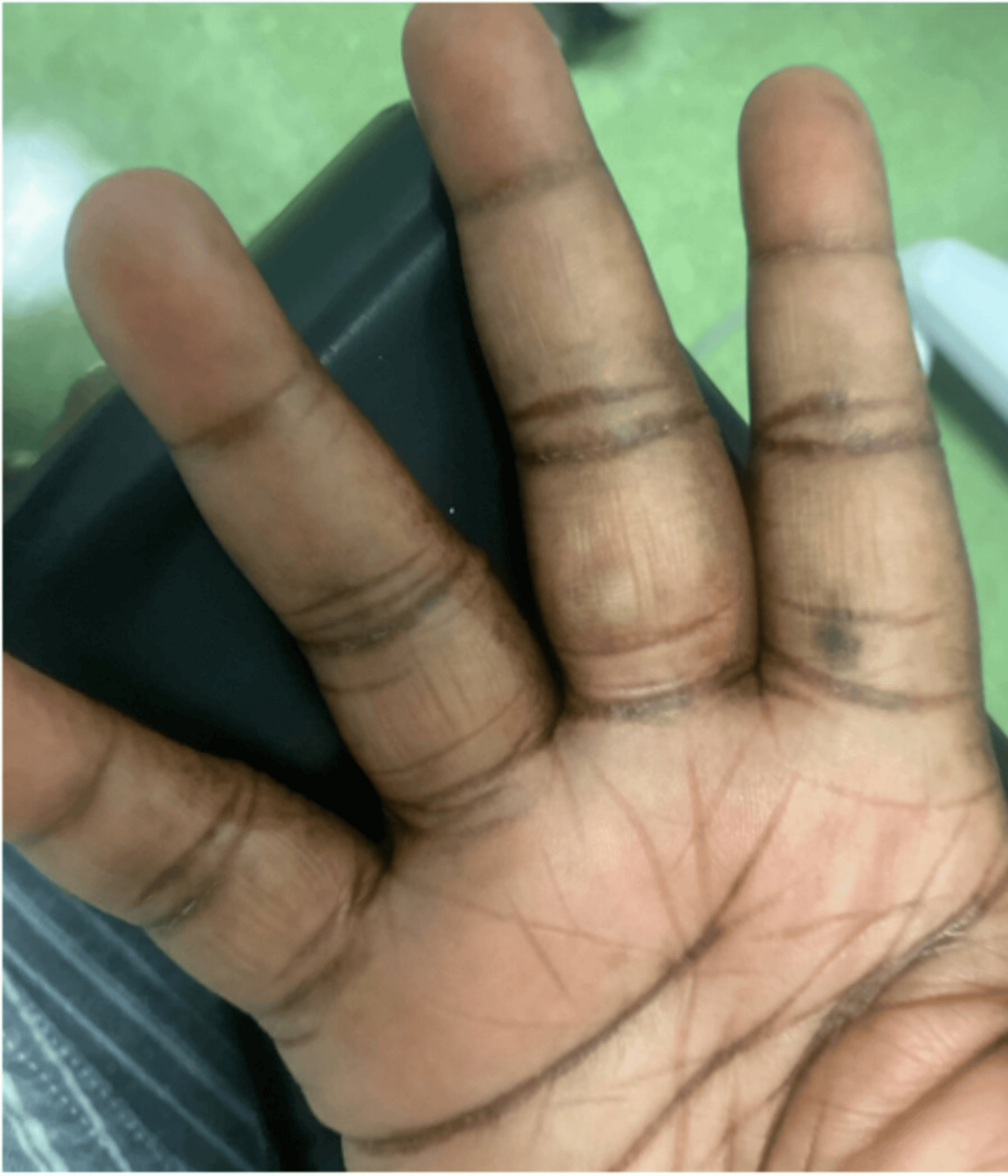
Right middle finger skin lesion with bluish nodular-type swelling

A 56-year-old female presented to the clinic due to a lesion located on the palmar aspect of her right middle finger. The patient had noted gradual growth of the area over the past year without associated pain or changes in color or borders (Figure 1).

The patient denied any systemic symptoms such as weight loss, fever, or fatigue. Her medical history included diabetes mellitus, gastric intestinal metaplasia, dyschromia, lichenoid dermatitis, and a trigger finger of the thumb. Past surgeries included drainage of a preauricular abscess, laparoscopic partial left salpingectomy, and cystectomy. Physical examination revealed a mildly mobile, bluish-in-color, nodular lesion on the palmar area of the right middle finger proximal phalanx, with slight tenderness on palpation.

**Figure 2:**
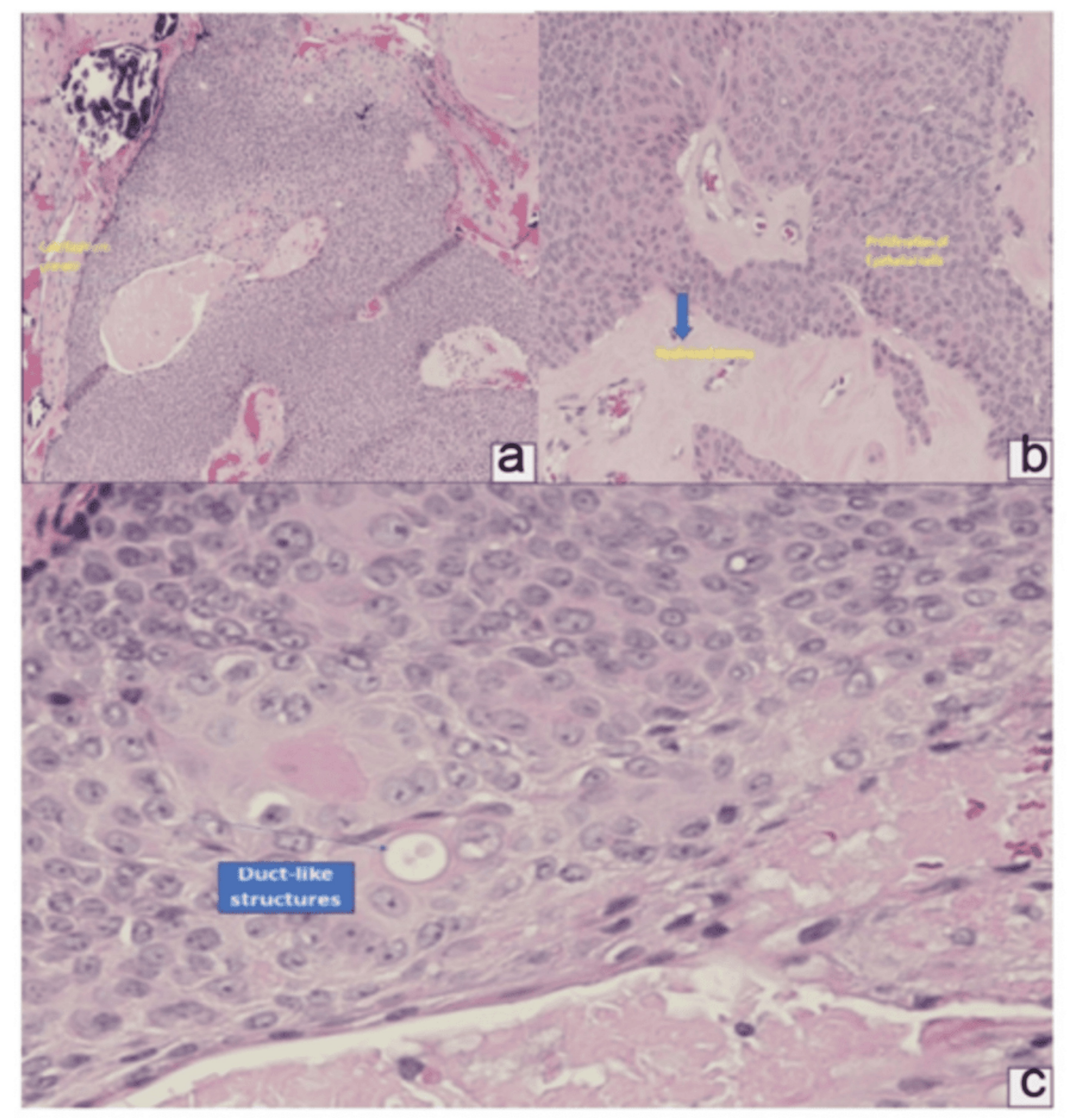
Histological findings a) H & E stain, X20. Sections show a non-encapsulated nodular proliferation of epithelial cells in the dermis. Calcifications present (blue arrows). b) A non-encapsulated nodular proliferation of epithelial cells in the dermis. The lesional cells are round and polygonal in shape with eosinophilic cytoplasm and focally basaloid, associated with hyalinized stroma (large blue arrow). Typical poroid cells with small round to streaming uniform nuclei, and pink cytoplasm (arrows). c) H&E stain, X40. The lesional cells are round and polygonal in shape with eosinophilic cytoplasm and focally basaloid, associated with hyalinized stroma. Cytologic atypia and mitosis are not prominent. Duct-like structures are also present (arrow)

The excision was done under local anesthesia. A 1 cm excision was made on the palmar surface encompassing the lesion. The lesion was carefully excised with protection of surrounding structures, and subsequently removed and sent for pathological analysis. The histological examination confirmed the diagnosis of a poroid hidradenoma. The specimen revealed a non-encapsulated nodular proliferation of epithelial cells in the dermis. The lesional cells were round and polygonal in shape with eosinophilic, pink cytoplasm, and small round to streaming uniform nuclei. The cells were focally basaloid and associated with hyalinized stroma. Cytologic atypia and mitosis were not prominent. Duct-like structures were also present (Figure 2).

## Discussion

Poroid hidradenoma is a rare tumor that originates from adnexal structures with eccrine features. Only one other report in the literature has cited this diagnosis as a finger lesion [5]. It belongs to the group of poroid neoplasms, accounting for fewer than 5% of all hidradenomas [6]. The broader category of hidradenomas encompasses both poroid and apocrine types, which share similar histomorphological characteristics, presenting challenges in their differentiation [7].

Studies in the literature have discussed the rarity of poroid hidradenoma in terms of developing into a malignant neoplasm. However, it remains extremely important to rule out any possible malignancies, such as digital papillary adenocarcinoma, before confirming the diagnosis of poroid hidradenoma. Studies indicate the importance of differentiating between digital papillary adenocarcinoma and benign cutaneous adnexal tumors such as a poroid hidradenoma, as both may be on acral sites and exhibit overlapping histological features [8]. Histological examination remains the standard diagnostic tool for poroid hidradenoma.

As with any finger lesion, patients may seek removal due to discomfort, aesthetic reasons, or concerns about malignancy. Surgical excision is the preferred treatment, and recurrence is rarely reported [4,9]. In this case, the lesion was surgically excised and sent to pathology, which provided the histological confirmation of the diagnosis. Overall, these tumors are classified as benign, given their low risk of malignant transformation, which is less than 1%. Moreover, the prognosis for poroid hidradenoma is generally favorable [3].

## Conclusions

Poroid hidradenoma, characterized by its eccrine differentiation and benign nature, may present as a solitary nodule causing discomfort or aesthetic concerns. In very rare cases, it may present as a finger lesion. Surgical excision remains the preferred treatment with a low risk of recurrence reported in the literature. Immunohistochemical studies are the key to confirming the diagnosis and they demonstrate the condition's similarities with eccrine poroma. Differential diagnosis includes other poromas, as well as benign subcutaneous connective tissue neoplasms such as fibroma, fibrolipoma, dermatofibroma, epidermal inclusion cyst, and papillary adenocarcinoma. Accurate histopathological assessment is essential for guiding appropriate management and treatment of skin lesions. Our case underscores the clinical and diagnostic challenges associated with poroid hidradenoma, as well as the importance of multidisciplinary evaluation to ensure optimal patient care and outcomes.
